# 

**Published:** 1907-09-21

**Authors:** 


### BOOKS RECEIVED.

Baillière, Tindall, and Cox.

"Nature's Hygiene and Sanitary Chemistry" (5th edition). By C. T. Kingzett, F.I.C.

A. Brown and Sons, Ltd.

"Modern Methods for Securing Surgical Asepsis." By Edward Harrison, F.R.C.S.

Rebman, Ltd.

"Medical Diagnosis." By C. L. Greene, M.D.

Surgery Publishing Co., New York.

"Five Hundred Surgical Suggestions." By W. M. Brickner, M.D., and Eli Moschcowitz, M.D.

J. Wright and Co.

"Consumption, Home Treatment, and Rules for Living." By H. Warren Crowe, M.D.

THE HOSPITAL *September* 21, 1907.

*Name* ..........................................................................................

*Address* ..........................................................................................

..........................................................................................

**This Coupon must accompany manuscript or contributions intended for THE HOSPITAL.**

